# Causal effects of genetically determined circulating metabolites on endometriosis: A Mendelian randomization study

**DOI:** 10.1097/MD.0000000000040690

**Published:** 2024-11-22

**Authors:** Lusha Liu, Junping Yin, Yakun Liu, Bin Li, Shan Kang, Naiyi Du

**Affiliations:** a Department of Gynecology, The Fourth Hospital of Hebei Medical University, Shijiazhuang, Hebei, China; b Department of Clinical Laboratory, Affiliated hospital of Hebei Engineering university, Handan, Hebei, China; c Department of Gynecology, Handan Central Hospital, Handan, Hebei, China.

**Keywords:** causal effect, endometriosis, Mendelian randomization, metabolites

## Abstract

Endometriosis (EMs) is a common gynecological disease accompanied by metabolic disturbances. However, the causality between metabolites and the risk of EMs remains unclear. We conducted a 2-sample Mendelian randomization (MR) analysis using the publicly available genome-wide association study (GWAS) of 486 circulating metabolites and EMs. The inverse variance weighted (IVW) was mainly used for assessing causality. MR–Egger intercept, MR-PRESSO Global, leave-one-out, and Cochran *Q* test analyses were used for sensitivity analyses. A total of 25 causal metabolites related to EMs have been identified, including 13 known and 12 unknown ones. Among the known metabolites, caffeine (OR = 0.86, 95% CI: 0.76–0.98, *P* = .026), cortisol (OR = 0.64, 95% CI: 0.41–0.99, *P* = .047), glycocholate (OR = 0.67, 95% CI: 0.51–0.87, *P* = .003), adrenate 22:4n6 (OR = 0.52, 95% CI: 0.35–0.77, *P* = .001), and ergothioneine (OR = 0.62, 95% CI: 0.47–0.81, *P* = .000) were protective factors for EMs, while mannose (OR = 1.43, 95% CI: 1.01–2.03, *P* = .044), 4-acetamidobutanoate (OR = 1.92, 95% CI: 1.27–2.89, *P* = .002), 1-linoleoylglycerol (OR = 1.36, 95% CI: 1.10–1.68, *P* = .005), bilirubin (Z, Z) (OR = 1.15, 95% CI: 1.01–1.31, *P* = .032), threonate (OR = 1.42, 95% CI: 1.14–1.77, *P* = .002), bilirubin (E, E) (OR = 1.18, 95% CI: 1.01–1.38, *P* = .039), erythronate (OR = 1.59, 95% CI: 1.01–2.52, *P* = .047), and dimethylarginine (SDMA + ADMA) (OR = 2.07, 95% CI: 1.19–3.62, *P* = .010) were risk factors for EMs. Additionally, there was no evidence of heterogeneity or pleiotropy of the known metabolites. Leave-one-out analysis indicated that the MR findings were robust. Our findings provide valuable circulating biomarkers as well as therapeutic targets for the screening, prevention, and treatment of EMs.

## 1. Introduction

Endometriosis (EMs) is a gynecological condition characterized by endometrial stromal and gland implantation outside the uterus with an unclear etiology, involving a range of factors including genetics, hormones, metabolism, and immunity.^[1]^ It affects approximately 190 million women of reproductive age worldwide.^[1]^ Symptoms like chronic pelvic pain and infertility severely compromise women’s physical and mental health. Due to the diversity of symptoms and the lack of definitive diagnostic markers in the early stages of the diseases, nearly 60% of female patients experience misdiagnosis.^[2]^ While current treatment options, such as surgery and medication, can help manage symptoms, they do not provide a cure for the disease. Therefore, further exploration of the etiology of endometriosis holds significant value for improving diagnostic and treatment strategies for this condition.

Metabolomics, as a key component of systems biology, focuses on analyzing the small-molecule metabolites (<1.5 kDa) present in bodily fluids.^[3]^ It reflects the biological mechanisms of diseases by revealing changes in intermediate metabolites and metabolic pathways.^[3,4]^ Interestingly, metabolomics studies related to EMs have shown varied results. For example, inconsistencies have been observed in the expression levels of acylcarnitine,^[5]^ lactate, 3-hydroxybutyrate, alanine, leucine, valine, threonine, lysine, glycerol phosphatidylcholine, succinic acid, as well as lipids, glucose, isoleucine, arginine,^[6]^ proline,^[7]^ and sphingomyelins^[8]^ in the blood of EMs patients across different studies. These findings suggest that changes in circulating metabolites may be vital for the development of EMs. Notably, discrepancies of these metabolomics findings may be attributed to differences in sample size, experimental techniques, and study subjects, potentially leading to the oversight of metabolites that play a crucial role in EMs. Recently, a database of genetically determined metabolites (GDMs) has been established through a nontargeted metabolomics-based genome-wide association study (GWAS), which helps to understand the potential relevance of human serum metabolites and their associated genetic variants in the pathogenesis of complex diseases.^[9–11]^ Based on the existing literature, we proposed the following hypotheses to guide this study, “Certain GDMs are causally associated with EMs; Some of these metabolites are protective and others are risk factors.” However, there are still challenges in translating these genetic discoveries into biological mechanisms of EMs development which needs to deep analysis to elucidate the causal interactions of serum metabolites on EMs susceptibility. In the current study, although GWAS has revealed the correlation between metabolites and a variety of complex diseases, there is no assessment of the causal effect of GDMs on EMs in the study of EMs. Therefore, the main objective of the present study was to fill this research gap by making the first attempt to assess the causal effect of GDMs on EMs through the Mendelian randomization (MR) method.

MR analysis is a powerful epidemiological tool that utilizes genetic variations as instrumental variables (IVs) to assess the potential causal relationship between exposures and outcomes.^[12]^ These IVs are determined at conception, thus helping to rule out the interference of confounding factors and reverse causality, thus rendering MR a more objective and precise tool in assessing causal effects compared to other methods.^[13]^ Therefore, based on the lack of causality assessment in current research on the relationship between metabolites and EMs, this study put forward the following 2 key research questions: “Which GDMs are causally associated with EMs? Among the metabolites that are causally associated with EMs, which are protective factors and which are risk factors?” This study provides the first systematic assessment of these questions through MR analysis, thus providing new insights into the noninvasive diagnosis and treatment of EMs.

## 2. Materials and methods

### 2.1. Study design

In the overall study design, we adopted the summarized data from the open database or published GWAS cohort (https://www.ebi.ac.uk/gwas/). The open database collected the GWAS summary data from published studies or the GWAS cohorts launched by some authoritative and qualified consortiums. By employing GWAS data, we can analyze a large number of samples with consistent methodologies, which helps to reduce variability and heterogeneity in the findings. The strength of using GWAS data lies in its ability to provide a comprehensive view of genetic associations with serum metabolites, allowing us to identify potential causal relationships more robustly. This approach minimizes the impact of confounding factors that may arise from differences in sample size, experimental techniques, and study subjects, ultimately leading to more reliable and generalizable results. The ethical approval of all these studies had been acquired by related review committees in their respective institutions. In this paper, we just utilized the summary data to deliver a 2-sample Mendelian randomization (TSMR) study for estimating the causal effect of 486 circulating metabolites on EMs. Therefore, no further sanction was demanded.

### 2.2. Data source of serum metabolites and endometriosis

The GWAS data for 486 circulating metabolites was obtained from a study by Shin et al.^[10]^ Using ultrahigh-performance liquid phase chromatography and gas chromatography coupled with tandem mass spectrometry, the authors analyzed the metabolic profiling of fasting serum samples from 7824 adult individuals in 2 European population studies. Eventually, the GWAS analysis included 486 metabolites and 8 comprehensive metabolic groups, with 177 categorized as “unknown.” Nevertheless, we incorporated these “unknown” metabolites into our analysis, as they persist as a subject of interest for researchers and may hold potential for providing valuable insights in future studies.^[14]^

The GWAS summary datasets for EMs were obtained from the open GWAS database. The GWAS ID is finn-b-N14_ENDOMETRIOSIS, with 77,257 female participants (8288 cases and 68,969 controls). All the data used in this study were obtained from publicly available GWAS summary statistics. No new data was collected, and no new ethical approval was needed.

### 2.3. Selection of instrumental variables

To obtain compelling MR results, the single nucleotide polymorphisms (SNPs) serving as IVs must satisfy 3 crucial assumptions: SNPs must be directly associated with the circulating metabolites; the effects of SNPs on EMs must be solely mediated by the circulating metabolites of interest; and SNPs must be completely independent of any potential confounders that may affect both circulating metabolites and EMs. Subsequently, the PhenoScanner V2 database (https://www.repository.cam.ac.uk/items/31e4df31-982b-452e-baf8-83ce942b1c0a) was used to verify all SNPs selected for examining potential violations of hypotheses (2) and (3). To minimize the bias effect on the eventual outcomes, SNPs relevant to the multiple confounding traits at the genome-wide significant level were stringently excluded. In our MR analysis, we selected IVs based on the 3 crucial assumptions outlined previously. Furthermore, we selected IVs with association thresholds of *P* < 1 × 10^−5^ for each metabolite. Next, we screened for independent IVs, using an *r*^2^ < 0.1 within a window size of 500 kilobase (kb), to assess linkage disequilibrium (LD). The SNPs related to exposure were excluded when they did not match the obtained GWAS statistical results. Finally, the proportion of variability (*R*^2^) and *F*-statistic were used to evaluate the strength of these IVs.^[15]^ Among them, *F*-statistics were calculated using the formula *F* = Beta^2^/SE^2^, and an *F*-statistic > 10 was used as a criterion for selecting IVs with strong effects^[16]^.

### 2.4. Statistical analysis

The “TwoSampleMR” package in R software-4.3.1 was used for all analyses.^[17]^ The primary statistical approach for inferring causality between metabolites and EMs was the inverse variance weighted (IVW) model,^[18]^ and the other MR methods such as weight median, weight mode, simple mode, and MR-Egger were served as an auxiliary method. Next, sensitivity analyses were then performed, utilizing MR-Egger intercept and MR-PRESSO Global test to assess pleiotropy, Cochran *Q* statistic to detect potential heterogeneity. When Cochran *Q* value indicated heterogeneity with a significance level of *P* < .05, the IVW estimation was recalculated using a random effects model. Furthermore, a leave-one-out analysis was conducted to assess the influence of individual SNPs on the overall results.

## 3. Results

### 3.1. Selection of IVs

A total of 10,540 SNPs correlated with the 486 metabolites were identified as IVs (Table S1, Supplemental Digital Content, http://links.lww.com/MD/O38). In addition, the *F*-statistics > 10 of the identified IVs indicates that they were informative for MR analysis.

### 3.2. Causal effects of metabolites on endometriosis

**Figure 1.**
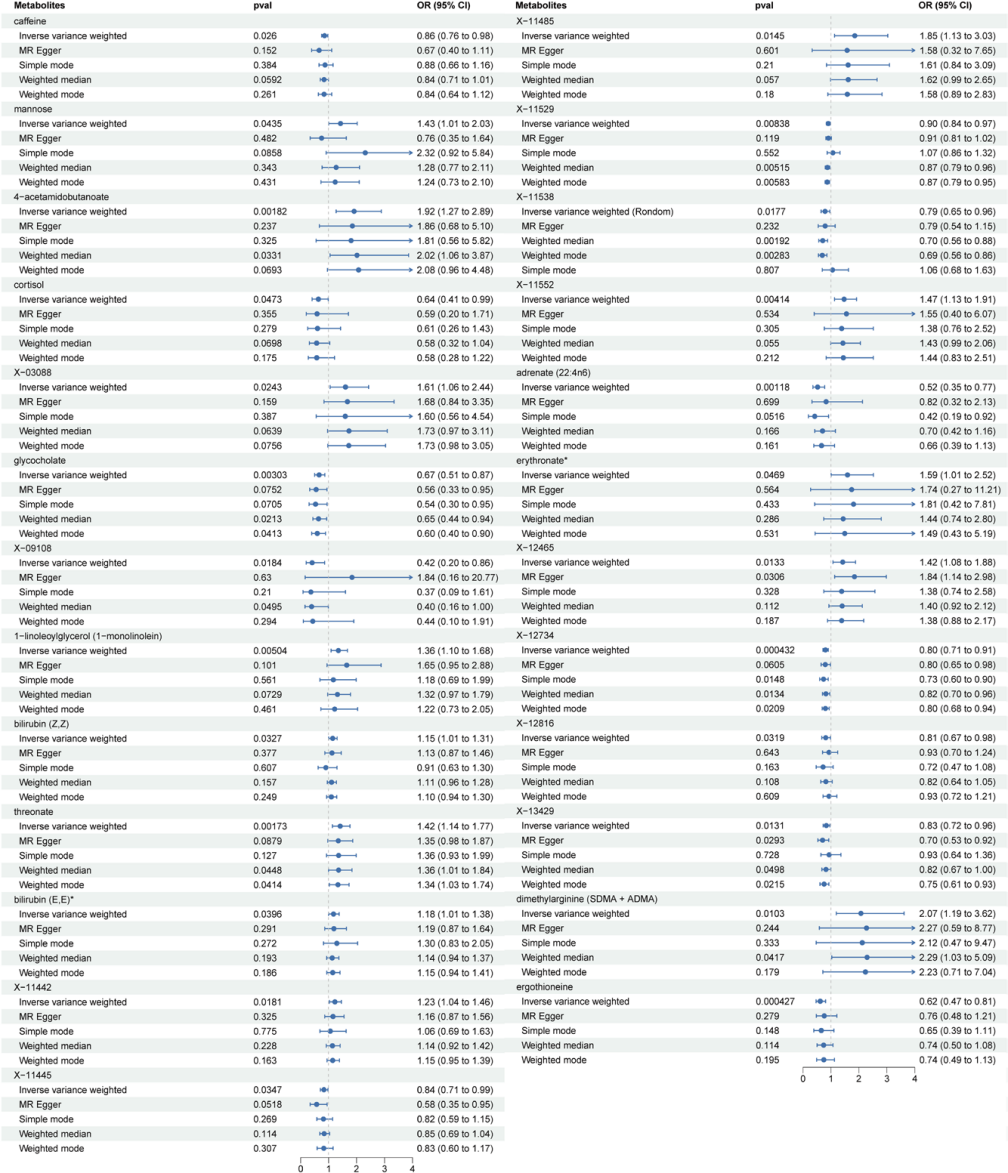
The forest plot of significant metabolites and the risk of endometriosis.

We identified 25 causality metabolites, including 13 known and 12 unknown ones, as shown in Figure 1 (Tables S2 and S3, Supplemental Digital Content, http://links.lww.com/MD/O38). In the 13 known metabolites, revealing that caffeine (OR = 0.86, 95% CI: 0.76–0.98, *P* = .026), cortisol (OR = 0.64, 95% CI: 0.41–0.99, *P* = .047), glycocholate (OR = 0.67, 95% CI: 0.51–0.87, *P* = .003), adrenate 22:4n6 (OR = 0.52, 95% CI: 0.35–0.77, *P* = .001), and ergothioneine (OR = 0.62, 95% CI: 0.47–0.81, *P* = .000) were negatively associated with EMs, suggesting a protective effect for EMs. Mannose (OR = 1.43, 95% CI: 1.01–2.03, *P* = .044), 4-acetamidobutanoate (OR = 1.92, 95% CI: 1.27–2.89, *P* = .002), 1-linoleoylglycerol (1-monolinolein) (OR = 1.36, 95% CI: 1.10–1.68, *P* = .005), bilirubin (Z, Z) (OR = 1.15, 95% CI: 1.01–1.31, *P* = .032), threonate (OR = 1.42, 95% CI: 1.14–1.77, *P* = .002), bilirubin (E, E) (OR = 1.18, 95% CI: 1.01–1.38, *P* = .039), erythronate (OR = 1.59, 95% CI: 1.01–2.52, *P* = .047), and dimethylarginine (SDMA + ADMA) (OR = 2.07, 95% CI: 1.19–3.62, *P* = .010) were positively linked with EMs.

### 3.3. Sensitivity analysis

**Figure 2.**
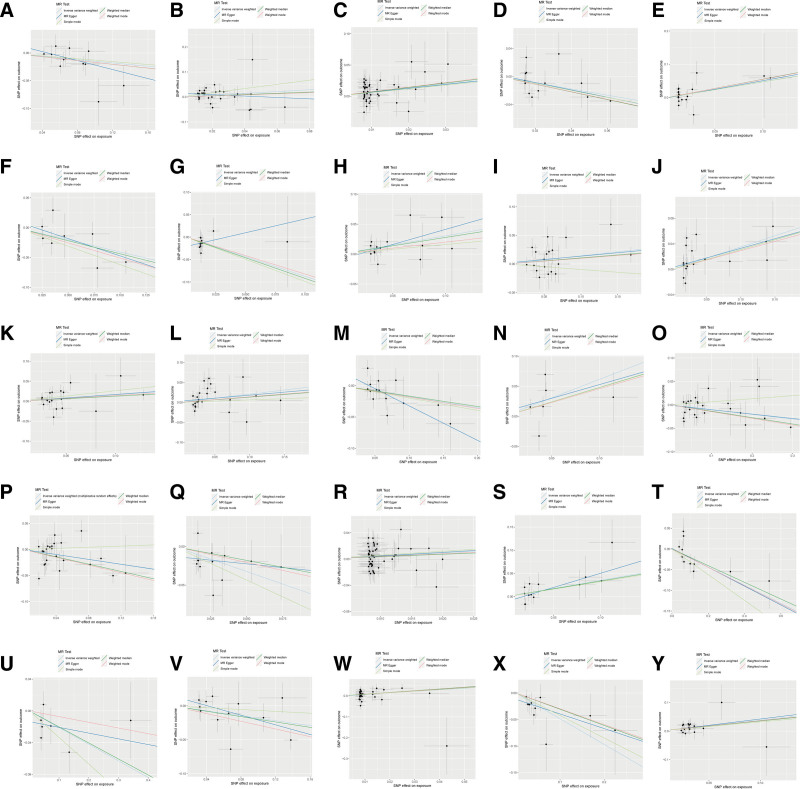
Scatter plots of significant metabolites associated with EMs. (A) Caffeine. (B) Mannose. (C) 4-Acetamidobutanoate. (D) Cortisol. (E) X-03088. (F) Glycocholate. (G) X-09108. (H) 1-Linoleoylglycerol (1-monolinolein). (I) Bilirubin (Z, Z). (J) Threonate. (K) bilirubin (E, E). (L) X-11442. (M) X-11445. (N) X-11485. (O) X-11529. (P) X-11538. (Q) Adrenate (22:4n6). (R) Erythronate. (S) X-12465. (T) X-12734. (U) X-12816. (V) X-13429. (W) Dimethylarginine (SDMA + ADMA). (X) Ergothioneine. (Y) X-11552. EMs: endometriosis.

**Figure 3.**
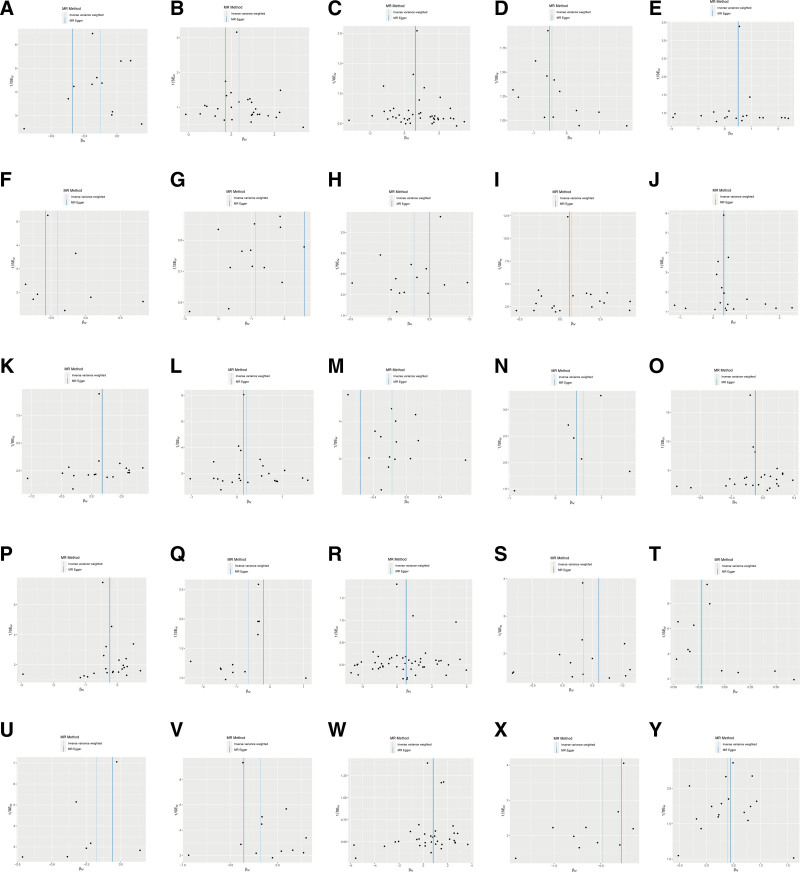
The funnel plot represents IVs for each significant causal association between metabolites and endometriosis. (A) Caffeine. (B) Mannose. (C) 4-Acetamidobutanoate. (D) Cortisol. (E) X-03088. (F) Glycocholate. (G) X-09108. (H) 1-Linoleoylglycerol (1-monolinolein). (I) Bilirubin (Z, Z). (J) Threonate. (K) Bilirubin (E, E). (L) X-11442. (M) X-11445. (N) X-11485. (O) X-11529. (P) X-11538. (Q) Adrenate (22:4n6). (R) Erythronate. (S) X-12465. (T) X-12734. (U) X-12816. (V) X-13429. (W) Dimethylarginine (SDMA + ADMA). (X) Ergothioneine. (Y) X-11552. IVs = instrumental variables

**Figure 4.**
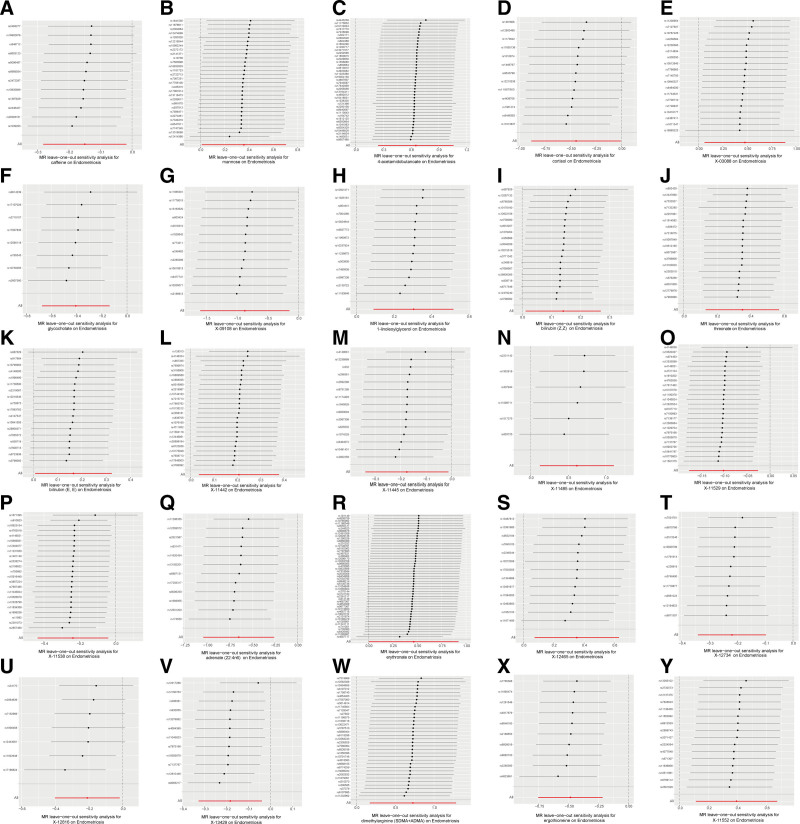
Leave-one-out analysis of the causal effects of metabolites and endometriosis. (A) Caffeine. (B) Mannose. (C) 4-Acetamidobutanoate. (D) Cortisol. (E) X-03088. (F) Glycocholate. (G) X-09108. (H) 1-Linoleoylglycerol (1-monolinolein). (I) Bilirubin (Z, Z). (J) Threonate. (K) Bilirubin (E, E). (L) X-11442. (M) X-11445. (N) X-11485. (O) X-11529. (P) X-11538. (Q) Adrenate (22:4n6). (R) Erythronate. (S) X-12465. (T) X-12734. (U) X-12816. (V) X-13429. (W) Dimethylarginine (SDMA + ADMA). (X) Ergothioneine. (Y) X-11552.

Specifically, as depicted in Table 1, sensitivity analyses were conducted to test the heterogeneity and horizontal pleiotropy of the selected IVs (Table S4, Supplemental Digital Content, http://links.lww.com/MD/O38). The results of MR-Egger intercept, MR-PRESSO Global test and scatter plot (Fig. 2) indicated that there was no horizontal pleiotropy or outliers. Cochrane *Q* test and funnel plot (Fig. 3) showed that no heterogeneity was observed except for X-11538. Thus, IVW of X-11538 were analyzed using random effects model. Leave-one-out analysis indicated that the MR findings were robust (Fig. 4).

**Table 1:** Evaluation of heterogeneity and pleiotropy of identified metabolites.

Metabolites	Heterogeneity test *Q* (*P*-value)	Pleiotropy (*P*-value)	
		MR-Egger	MR-PRESSO
Caffeine	.55	.34	.58
Mannose	.21	.08	.24
4-Acetamidobutanoate	.76	.95	.76
Cortisol	.36	.87	.41
X-03088	.23	.88	.32
Glycocholate	.33	.48	.42
X-09108	.95	.23	.96
1-Linoleoylglycerol	.63	.46	.63
Bilirubin (Z,Z)	.12	.85	.16
Threonate	.23	.69	.34
Bilirubin (E,E)	.25	.94	.32
X-11442	.15	.65	.17
X-11445	.22	.15	.22
X-11485	.06	.84	.14
X-11529	.40	.85	.41
X-11538	.04	.98	.051
X-11552	.92	.94	.93
Adrenate (22:4n6)	.35	.32	.42
Erythronate	.26	.92	.26
X-12465	.42	.23	.49
X-12734	.28	.92	.42
X-12816	.35	.27	.39
X-13429	.31	.18	.26
Dimethylarginine (SDMA + ADMA)	.37	.89	.39
Ergothioneine	.81	.31	.83

## 4. Discussion

Endometriosis (EMs) is increasingly recognized as a disease related to metabolism, not only because of its association with various metabolic disorders, but also due to the discovery of numerous abnormal metabolites in EMs metabolomics studies. Blood, rich in detectable metabolites and easily accessible, serves as an important source for metabolomic identification. Previous plasma/serum metabolomics studies have revealed alterations in the metabolic profiles of EMs patients.^[19–21]^ For example, Maignien et al conducted a serum metabolomics analysis of EMs using ^1^H-NMR, which revealed decreased concentrations of glutamine, valine, threonine, histidine, tyrosine, and isoleucine, as well as increased concentrations of free fatty acids in EMs patients compared to those not affected.^[19]^ Murgia reported that, in endometriosis patients, β-hydroxybutyric acid and glutamine levels were significantly increased, whereas tryptophan levels were significantly decreased.^[20]^ Additionally, Loy et al conducted a peritoneal fluid metabolomics analysis between peritoneal endometriosis and controls and further validated in serum of patients, which serum phenylalanyl-isoleucine could distinguish peritoneal endometriosis from controls.^[21]^ Unfortunately, although observational studies can be used to investigate the relationship between diseases and phenotypes, they cannot assess causality. Herein, based on GWAS data of metabolites and EMs, we conducted a 2-sample MR analysis to evaluate the causal relationship between metabolites and EMs. We identified 25 metabolites associated with the risk of EMs, including 13 known metabolites and 12 unknown ones. Among the known metabolites, caffeine, cortisol, glycocholate, adrenate 22:4n6 and ergothioneine were negatively correlated with EMs, indicating that they may be protective factors. On the other hand, mannose, 4-acetamidobutanoate, 1-linoleoylglycerol (1-monolinolein), bilirubin, threonate, bilirubin (E,E), erythronate, and dimethylarginine (SDMA + ADMA) were positively correlated with EMs, suggesting that they may be risk factors. Notably, our study emphasizes the significance of metabolites could play a vital role in the pathological mechanisms of EMs.

### 4.1. Protective factors

Compared to previous studies, our findings are both consistent and novel. Caffeine has been found to impact hormone-dependent diseases by regulating the levels of steroid hormones and the production of sex hormone-binding globulin (SHBG) in the liver, as well as influencing aromatase function in the conversion of androgens to estrogens.^[22–24]^ Current evidence on the relationship between caffeine and the risk of EMs remains inconclusive based on existing observational studies.^[25–28]^ Specifically, some studies suggest a potential risk of caffeine on EMs,^[25,26]^ while others indicate no significant association.^[27,28]^ The variability in study designs, sample sizes, and populations, which may contribute to these conflicting results. A meta-analysis by Kechagias et al indicated that caffeine consumption does not seem to be linked to an increased risk of EMs. However, further analysis revealed that high caffeine intake (>300 mg/day) was associated with an elevated risk of EMs.^[29]^ Some studies have demonstrated that caffeine can inhibit aromatase activity and increase secretion of SHBG,^[30,31]^ potentially reducing estrogen bioavailability and lowering the risk of EMs. This finding supports our result indicating that caffeine might have a protective effect against EMs, although the exact biological mechanism underlying this association requires confirmation through experimental studies. Cortisol, a stress hormone produced by the fasciculate zone of the adrenal cortex, is essential for maintaining body homeostasis and exhibits anti-inflammatory properties at physiological levels.^[32,33]^ Several studies have indicated decreased cortisol levels in different EMs test samples,^[33,34]^ implying a potential protective role of cortisol in EMs. Our findings are consistent with these reports, but further research is needed to validate the underlying mechanism. Ergothioneine (EGT), a histidine derivative containing sulfur, has been widely studied as a potent antioxidant for many years.^[35]^ Possibly due to its antioxidant role, our analysis revealed that EGT could be a protective factor against EMs. Furthermore, our study sugested that adrenate (22:4n6) and glycocholate are protective factors against EMs.

### 4.2. Risk factors

Mannose, a natural bioactive monosaccharide, plays a crucial role in modulating metabolic and inflammatory processes. The mannose receptor (MR, CD206) is essential for maintaining mannose functionality and is involved in the recognition and phagocytosis of antigens.^[36]^ MR^+^ dendritic cells (DCs) facilitate the phagocytosis of dead endometrial cells, leading to increased expression of IL-1β and IL-6, cytokines that contribute to the development of EMs.^[37]^ Moreover, CD206^+^ macrophages upregulate the expression of TGFβ1 and VEGFA, promoting angiogenesis and the formation of endometriotic lesions.^[38]^ Our findings suggest a positive association between mannose and the risk of EMs, with the underlying mechanism potentially involving the regulation of inflammation and immune responses related to EMs through the action of mannose on MR. Bilirubin, an endogenous antioxidant, is intricately involved in the metabolism of hemoglobin and prophyrin.^[39]^ Existing findings suggest that bilirubin has immunosuppressive effects on antigen-presenting cells and T cells.^[40,41]^ Currently, the relationship between bilirubin levels and EMs remains a topic of debate among researchers. Some studies report no significant difference in bilirubin levels between women with and without EMs,^[42,43]^ while others indicate higher levels of bilirubin in EMs patients compared to controls.^[44,45]^ Our own research supports this, showing a positive correlation between bilirubin levels and the risk of EMs. We speculated that elevated bilirubin levels may have an immunosuppressive effect that contributes to the development of EMs. Our study revealed a positive correlation between dimethylarginine (SDMA and ADMA) and the risk of EMs. Dimethylarginine is composed of 2 structural isomers, symmetric dimethylarginine (SDMA) and asymmetric dimethylarginine (ADMA), both of which act as endogenous inhibitors of nitric oxide synthase (NOS), leading to endothelial dysfunction and contributing to various diseases.^[46,47]^ Kinugasa et al found that patients with EMs had significantly higher plasma levels of ADMA and inflammatory markers compared to healthy individuals.^[48]^ Meanwhile, inflammation could also elevate ADMA levels in patients with EMs.^[49]^ These results suggest that dimethylarginine may be involved in endothelial dysfunction of EMs. Erythronate, an organic acid, can function as an oxidative stress product.^[50]^ 4-Acetamidobutanoate, a urea cycle product, is associated with decreased function in liver and kidney disease. Threonate often acts as a Mg2^+^ ionophore. According to our results, the 3 abovementioned 3 metabolites and 1-linoleoylglycerol are potentially harmful factors for EMs. It is important to note that the underlying mechanisms responsible for the effects of these metabolites on EMs are not fully understood, highlighting the need for further investigation in future studies.

This study has several limitations. First, the data on metabolites were primarily sourced from European populations; therefore, caution should be taken when generalizing the findings to other ethnic groups. Second, although this study successfully identified several metabolites associated with EMs risk, our understanding of the precise functions and mechanisms underlying certain metabolites relating to EMs is limited. Third, as MR analysis is a hypothesis-driven approach, additional experimental and clinical studies are necessary to validate these findings more comprehensively. Ultimately, the exact causal relationship between different variables was actually based on “genetic liability,” which would be easily affected if IV data were not properly treated. Therefore, in future research, increasing the sample size to enhance statistical power and improve the reliability of the results, as well as conducting multi-site studies to improve the generalizability of findings across different populations and settings, may address these limitations.

## 5. Conclusions

In summary, this MR study firstly evaluated the causality between 25 serum metabolites and EMs, which are classified into protective factors and risk factors. Our findings not only offer novel insights into the underlying mechanisms of metabolites in EMs development but also provide valuable information for utilizing circulating metabolites as diagnostic and therapeutic biomarkers of EMs.

## Author contributions

**Conceptualization:** Lusha Liu, Naiyi Du.

**Data curation:** Lusha Liu, Junping Yin.

**Methodology:** Yakun Liu.

**Project administration:** Shan Kang.

**Software:** Bin Li.

**Supervision:** Naiyi Du.

**Visualization:** Junping Yin, Yakun Liu.

**Writing – original draft:** Lusha Liu, Junping Yin, Bin Li.

**Writing – review & editing:** Shan Kang, Naiyi Du.

## Supplementary Material


